# Cost-effectiveness of Cerebrolysin as an add-on treatment for neurorecovery after traumatic brain injury

**DOI:** 10.25122/jml-2025-0087

**Published:** 2025-04

**Authors:** Stefan Strilciuc, Diana Alecsandra Grad, Cristian Vlădescu, Anca Dana Buzoianu, Carmen Albu, Dafin Fior Mureșanu

**Affiliations:** 1 Department of Genomics, MEDFUTURE Institute for Biomedical Research, Iuliu Hațieganu University of Medicine and Pharmacy, Cluj-Napoca, Romania; 2 RoNeuro Institute for Neurological Research and Diagnostic, Cluj-Napoca, Romania; 3 National Institute for Management of Health Services, Bucharest, Romania; 4 Faculty of Medicine, Titu Maiorescu University, Bucharest, Romania; 5 Department of Clinical Pharmacology, Iuliu Hatieganu University of Medicine and Pharmacy, Cluj-Napoca, Romania; 6 Department of Neurology, University of Medicine and Pharmacy, Craiova, Romania; 7 Department of Neurology, Neuropsychiatry Hospital, Craiova, Romania; 8 Department of Neuroscience, Iuliu Hatieganu University of Medicine and Pharmacy, Cluj-Napoca, Romania

**Keywords:** Cerebrolysin, mild traumatic brain injury, cost-effectiveness analysis, functional outcome, anxiety, depression

## Abstract

Traumatic brain injuries (TBIs) are a leading cause of death and long-term disability worldwide, with incidence and injury mechanisms varying by age group and region. Impairment of functional status, diagnoses of anxiety and depression are encountered post-TBI. Studies have shown that Cerebrolysin can have positive effects among TBI survivors. We conducted a cost-effectiveness analysis (CEA) among patients with moderate TBI, using data from the CAPTAIN II trial. This exercise was carried out on a three-month timeline from the provider’s perspective. Two models were incorporated in the CEA: control (placebo group) and treatment (Cerebrolysin group). Our analysis showed that Cerebrolysin had a high probability of being cost-effective, based on Glasgow Outcome Scale Extended (GOSE) (in over 80% of patients with moderate TBI), Hospital Anxiety and Depression Scale (HADS) Depression and Anxiety scores (for the former two, in over 95% of patients with moderate TBI), when assuming a lasting effect (12 months) of the CAPTAIN trial intervention protocol. A model-based approach is needed to account for potential sources of bias beyond the 90-day observation period of this clinical trial. Furthermore, economic evaluations incorporating patients diagnosed with all TBI severities are needed.

## INTRODUCTION

The increasing burden of traumatic brain injury (TBI) on societies and economies requires concentrated efforts at the local, regional, and national levels to prevent its occurrence and mitigate adverse health outcomes among surviving patients [1,2]. With an estimated 49 million cases of TBI worldwide (95% confidence interval [CI]: 47–51 million) and 7.1 million years lived with disability (YLDs; 95% CI: 5.0–10 million), TBI ranks as the fourth leading cause of disabling neurological disorders globally [3]. In Romania, TBI has consistently ranked among the top three causes of disability for people over 25 years of age (ranking maintained between 1990 and 2019), with incidence nearly twice as high in men as in women, according to the World Health Organization (WHO) Rehabilitation Need Estimator [4].

Neurorehabilitation after hospital discharge must be individualized to each patient’s specific deficits and address the broad spectrum of post-TBI sequelae, such as motor and speech impairments, depression, anxiety, and post-traumatic stress disorder (PTSD)[5-8]. To meet these substantial unmet needs, pharmacological and non-pharmacological interventions are essential for reducing the overall burden of TBI-related impairments [9,10]. During the first year post-TBI, anxiety was reported in 75.7% of patients with moderate or severe TBI, and major depressive disorder among 51.6% of patients with moderate TBI and 57.1% with severe TBI [11,12]. Moreover, a multicenter, multinational study found that, at 3 months post-injury, rehabilitation services remain underprescribed: unmet needs ranged from 66% for physiotherapy to 37% for occupational and cognitive therapy, and speech therapy was recommended for only 39% of patients [13].

Cerebrolysin is a widely used pharmacological intervention to promote neurorecovery after TBI, as an add-on to standard clinical protocols. Clinical studies in moderate and severe TBI populations have demonstrated that Cerebrolysin improved patients’ health and rehabilitation process, supporting its inclusion among reimbursed drugs to enhance accessibility across socioeconomic groups [14,15]. Moreover, a recent systematic review and meta-analysis of 27 studies focusing on the effect of Cerebrolysin administered in varying doses and treatment durations, in patients with mild to severe TBI, provided additional evidence on the positive effects in patients with varying severities [16].

In Romania, however, many TBI survivors struggle to regain their pre-injury level of functioning due to limited access to recommended rehabilitation services [17]. Contributing factors include lengthy waiting lists, shortages of trained therapists and specialized equipment, excessive patient loads in outpatient clinics, and restrictive reimbursement policies that often cover only a small fraction of necessary therapies, particularly for those with more severe injuries [18-21].

Considering that at the European Union (EU) level Romania ranks second lowest in health expenditure per capita (measured in purchasing power parity [PPP]) and lowest in health spending as a share of gross domestic product (GDP), and that it faces a high burden of noncommunicable diseases and declining vaccination rates, cost-effectiveness analyses are essential tools to support policymakers in making reimbursement decisions for two or more interventions targeting the same diagnostic(s) considering constrained health system budgets [22–26].

Several economic evaluations of Cerebrolysin have been performed in Russia and Austria for patients with stroke [27], such as cost-effectiveness analyses comparing Cerebrolysin to standard treatment [28], combined cost-effectiveness assessments of Cerebrolysin plus alteplase versus alteplase alone [29], and budget impact analyses across different severity levels [30]. However, no country-specific economic evaluations exist for Cerebrolysin in Romanian patients with TBI, despite the critical importance of local cost-effectiveness data for national reimbursement decisions [31]. To address this gap, we performed a cost-effectiveness analysis (CEA) of Cerebrolysin in moderately severe TBI, based on a secondary data analysis of the CAPTAIN II trial, conducted in Romania.

## MATERIAL AND METHODS

We employed probabilistic methods for the following cost-effectiveness exercise and computed the incremental cost-effectiveness ratio (ICER) for Cerebrolysin in patients with moderate TBI. Our analysis is based on data retrieved from the CAPTAIN II trial, which aimed to assess the efficacy and safety of Cerebrolysin in patients diagnosed with moderate or severe TBI [14]. The CAPTAIN II study was a randomized, placebo-controlled, prospective, double-blind clinical trial conducted in a single center. Based on the inclusion/exclusion criteria (further detailed in the online protocol added to the ISRCTN registry [32]), patients with TBI (over 18 years old, with a Glasgow Coma Scale between 7 and 12) were allocated to either the treatment group or the control group. Patients allocated to the first group were administered 50 ml of Cerebrolysin (diluted in 0.9% NaCl/250 ml) at the first visit (days 1-10) or 10 ml of Cerebrolysin at two subsequent visits (days 31-40 and 61-70) [32].

Cost data was collected by the principal investigator, using patient identification numbers, which were previously provided after the patient signed the informed consent. Therefore, an additional IRB approval was not necessary to query the hospital's administrative database. From the CAPTAIN II trial database, we obtained patient demographics (age, gender), study arm allocation (Cerebrolysin or placebo), admission Glasgow Coma Scale (GCS) score, and outcome measures recorded at the third visit (day 90): Extended Glasgow Outcome Scale (GOSE) and the two subscales of the Hospital Anxiety and Depression Scale (HADS-Anxiety and HADS-Depression). At the treatment visits, patients from both study groups were evaluated according to the study protocol that defined primary and secondary outcome measures [32]. We conducted a descriptive analysis to confirm that treatment and control groups were comparable at baseline with respect to age, gender distribution, and TBI severity.

For this economic analysis, we aimed to assess the cost-effectiveness of Cerebrolysin on global status (evaluated with GOSE) and anxiety and depression (evaluated with HADS). GOSE is used to evaluate the global outcome in patients following a traumatic brain injury. It contains 19 items grouped in assessment domains: consciousness, function in the home, function outside the home, work/study, social and leisure activities, family and friendships, and symptoms. The scale categorizes the results into eight outcomes: death (1), vegetative state (2), disability (lower severe – 3, upper severe – 4, lower moderate – 5, and upper moderate – 6), and recovery (lower good – 7, upper good – 8)[33]. A revised version can also be used for the diagnosed pediatric population with TBI [34,35].

HADS is composed of two subscales, each having seven questions. One subscale measures anxiety, and the other, depression. The obtained scores can be interpreted as mild severity (ranging between 8 and 10), moderate (ranging between 11 and 14), and severe (ranging between 15 and 21) [36,37]. This scale has been validated in patients with different diagnoses (including TBI) and multiple languages [38–42].

This analysis focused on the moderate-TBI subgroup (*n* = 109) with complete GOSE and HADS data at day 90 (60 patients in the Cerebrolysin arm and 49 in the placebo arm). Severe-TBI patients were excluded to preserve statistical power [43]. We collected data on hospital costs and length of stay for patients from both study groups who were hospitalized following the traumatic brain injury. The total cost for each patient was composed of the following items: hospitalization (which was calculated according to the following formula: number of days x tariff/number of days), food, medicine, medical supplies, lab tests, and other investigations. We retrieved the cost for Cerebrolysin and saline solution from CANAMED (The National Catalog of Prices for Medicine for Human Utilization Provided with Medical Prescription, and Market Authorized) [44]. Treatment administration in an outpatient setting was calculated as an average tariff/service for five years (2013–2017) during which patients were enrolled. All costs recorded in Romanian lei (RON) were converted to euros (EUR) using the National Bank of Romania exchange rate (1 EUR = 4.9315 RON) [45], and we applied a 3% annual discount rate for hospitalization, treatment, and their administration in outpatient settings. Incremental cost-effectiveness ratios (ICERs) were calculated as the additional cost per one-point gain in QALYs (derived from GOSE utilities) and per one-point improvement in HADS-Anxiety and HADS-Depression.

Considering the methodological limitations of the collected trial data, we used the corresponding QALYs for each GOS-E score, as reported by Behranwala *et al*. [46] (GOS-E 1: 0.00, GOS-E 2: 0.11, GOS-E 3: 0.41, GOS-E 4: 0.58, GOS-E 5: 0.70, GOS-E 6: 0.81, GOS-E 7: 0.86, GOS-E 8: 1.00), estimated for a horizon of 1-year and divided the final values by four (in order to consider the duration of the CAPTAIN II trial for one patient – 90 days).

We conducted this trial-based cost-effectiveness analysis from the provider’s perspective, based on the timeline of the CAPTAIN II study (90 days). We calculated the ICER values only for a clinical trial scenario, for the groups of patients with traumatic brain injury from the control and treatment arms of the randomized controlled trial. We performed a probabilistic sensitivity analysis (PSA) to account for the uncertainty of the employed model, and, in an Excel workbook, we simulated 1,000 ICER scenarios for each of the instruments of interest. When interpreting the results of PSA, a typical example of cost-effectiveness threshold (50.000 EUR cost per QALY) over 12 months was multiplied by four to reflect the limited duration of our analysis (3 months), thus assuming that intervention effects remained linear and stable after the completion of the CAPTAIN treatment regimen.

## RESULTS

### Descriptive statistics

The sample for this cost-effectiveness exercise comprised mostly male patients with moderate TBI. 55.05% of the sample was composed of patients included in the treatment group. Both the treatment and the control groups were predominantly male patients (90% in the treatment group and 87.77% in the control group). The average GCS at admission was 10.7 (range: 9–12) in the treatment group and 10.91 (range: 9–12) in the control group.

On day 90, GOSE scores ranged from 5 to 8 in both the Cerebrolysin and placebo groups. HADS-Anxiety scores ranged from 0 to 16 in the Cerebrolysin arm and 0 to 17 in the placebo arm; HADS-Depression scores ranged from 0 to 15 and 0 to 18, respectively. The difference in average scores between study groups was -1.322 for HADS-Anxiety (Cerebrolysin: 6.364 vs. Placebo: 7.686), and 1.355 for HADS-Depression (Cerebrolysin: 5.788 vs. Placebo: 7.143). The difference between QALYs was 0.002 (Cerebrolysin: 0.236 vs. Placebo: 0.234).

### Cost and cost-effectiveness results

The total cost difference between study groups was 171,529.1 RON (38,783.7 EUR), with higher costs incurred by the treatment group. On a patient level, the cost for a patient from the placebo group was 2103.9 RON (431.2 EUR) lower than in the intervention group. The cost of treatment for the Cerebrolysin group sessions was 2206 RON (447.33 EUR) and 99 RON (20.1 EUR) for the control group.

**Figure 1:**
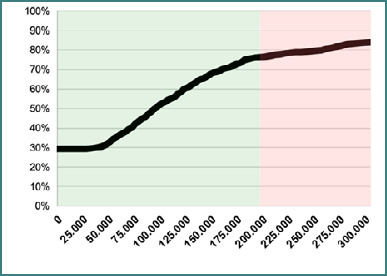
Cost-effectiveness acceptability curve (CEAC) for QALYs (based on corresponding utilities for GOS-E). Green and red shading highlights a cost-effectiveness threshold of 50.000 EUR per QALY, drawn from the extrapolation of treatment effects to a 12-month timeframe.

In the clinical trial scenario, for the GOSE scale, the Cerebrolysin add-on treatment was cost-effective in over 80% of patients with moderate TBI (Figure 1). As for HADS-Anxiety and HADS-Depression, Cerebrolysin was deemed effective in over 95% of patients.

The resulting deterministic ICERs were 2907 RON (589.531 EUR) per one-point improvement in HADS-Anxiety and HADS-Depression, and 63944 RON (12.966 EUR) for one point per QALY.

## DISCUSSION

To our knowledge, this is the first cost-effectiveness analysis of Cerebrolysin in patients with TBI, focusing on the global outcome and some of the most common post-TBI sequelae: depression and anxiety [6]. This study is relevant as it explores the cost-effectiveness of Cerebrolysin from a provider perspective and represents a starting point for conducting additional economic evaluations needed to inform policymaking decisions related to the allocation of financial resources for interventions with the best value for money [31].

Cerebrolysin is already approved for clinical use in Europe, Asia, and South America for vascular dementia, Alzheimer’s disease, stroke, and TBI [15]. Ghaffarpasand *et al*. highlighted several aspects regarding clinical studies, mainly that the encountered heterogeneity is caused by different inclusion criteria (either focusing on patients with one severity level or patients pertaining to several severity groups); this aspect is also the cause of a decreased study power. In the meta-analysis of studies published between 2005 and 2017, Cerebrolysin treatment was associated with significantly higher Glasgow Outcome Scale (GOS) scores and lower modified Rankin Scale (mRS) scores versus control groups [47]. A recently published meta-analysis and systematic review by Jarosz *et al*. [16] included studies published until July 2022 (*n* = 10) and analyzed the reported results on two scales (GOS and GCS) as well as two hospital-related indicators (mortality and length of stay). The age of the TBI patients from the included studies ranged between 30 and 47.4 years, and most patients were male (percentage sample ranging from 62 to 92). The duration of the included trials was 28 days to 6 months (and doses per day ranged from 10 to 50 mL/day, considering patient severity and pathology). The results of the meta-analysis showed that TBI patients had improved outcomes (in terms of functions measured using GOS and GCS), while morbidity and length of stay were not affected by the treatment with Cerebrolysin [16]. As for depression and anxiety, the existing information is currently insufficient to draw robust conclusions. Trials with a higher number of participants, as well as comparative-effectiveness analyses, are needed to reinforce or contend with existing outputs in a broader clinical setting [14,48-51].

Health Technology Assessment (HTA), including cost-effectiveness analyses alongside other economic evaluations and clinical studies, is essential for guiding resource-allocation decisions within health systems [22,52]. In Romania, a formal HTA process was introduced in 2013 through a score-card methodology that integrates the opinion of agencies from other countries (for example, from France and the United Kingdom), safety and patient-reported outcome data, and the number of EU countries that reimburse a given therapy [53]. Recently, a revised score-card system for HTA has addressed other issues such as budget impact considerations and encouraging the use of local data, yet major criticisms remain regarding the absence of national economic evaluation exercises, which should capture Romania's unique patient pathways [54]. In a 2016 comparative analysis of HTA transparency in Hungary, Romania, and Turkey, researchers found that Romania routinely missed its legislated three-month deadline for final report publication and failed to specify a formal reimbursement date. They also noted that key economic evaluation components—particularly budget-impact analyses—remained poorly defined [55], a shortcoming that has persisted since the inception of the HTA system within the national context [51]. A survey published in 2018 on the implementation pathway on HTA in Romania reported that HTA specialists would add other specifications for the HTA processes related to producing evidence (adding academic entities to the process; incorporating local data, soft thresholds, therapeutic values and cost-effectiveness studies; investing in patient registries; encouraging the use from payer databases)[56]. The Ministry of Health has contracted technical assistance to deal with this issue by reforming the HTA system from the ground up. In this context, the emergence of cost-effectiveness studies such as the one reported in this manuscript is an important stepping stone to jump-starting the development of national economic evaluations.

Our analysis indicates Cerebrolysin, when provided under the CAPTAIN II protocol (post-TBI days 1-10 – 50ml/day; days 31-40 – 10ml/day; day 61-70 – 10ml/day) is cost-effective if assuming treatment effects will remain constant beyond the 90-day observation period of the trial, up to 12 months after TBI. Nevertheless, our secondary analysis has several limitations that should be discussed. First, it lacks primary data on health-related quality of life [57], required to estimate quality-adjusted life years (QALYs), a commonly used and recommended measure that should be incorporated in economic evaluations [58]. The Romanian value set for the EQ-5D-5L tool was published in 2022 [59], after the conclusion of the CAPTAIN II trial in December 2017 [32]. We used the corresponding values for QALYs for GOSE (as reported by Behranwala *et al*. [46]); nevertheless, a mapping exercise (when a start measure is mapped into a target measure using different types of regressions) was not performed. Secondly, as our study only uses secondary data from a clinical trial, it fails to account for outcomes beyond its duration of the trial (90 days) and thus, provides estimates only on a short-term basis (although it is recommended that ICERs should be computed between 2 or 10 years or employing a lifetime horizon). Furthermore, the sample was composed only of patients diagnosed with a moderate form of TBI, and the sample size was relatively small, which reduces the power of the analysis [43].

Understanding the use of cost-effectiveness thresholds in this manuscript is important for correctly interpreting our results. Given the limited follow-up timeframe in the CAPTAIN II trial (90 days, or approximately 3 months), it is challenging to interpret cost-effectiveness analyses under a cost per QALY threshold, which is usually associated with an entire reference year (12 months). Therefore, we assumed that intervention effects would remain static even after study protocol completion, by multiplying the reference threshold of 50.000 EUR four times. This would have been equivalent to multiplying the amount of QALYs for both study groups under the same threshold. While this approach was the only one feasible given the inputs available to us at the time of analysis, a modeling approach (either Markov, discretely integrated condition events, etc.) is required to avoid important biases resulting from the potential changes in the utilities of TBI survivors in the 4–12-month period, such as complications, new post-traumatic mental health disorders and others. Readers should therefore exercise caution when interpreting the results, particularly in the context of limited international guidelines available for this purpose. Moreover, as our cost-effectiveness analysis was conducted for the timespan of the CAPTAIN II trial, other limitations are represented by the fact that recruitment occurred in a county, urban, academic hospital, and by a restrictive patient inclusion/exclusion checklist. Due to the perspective employed–the one of the provider–and also due to the nature of the data available, we did not include non-medical costs (i.e., productivity-related costs for both the patient and their family, as well as paid caregiver). Finally, we excluded participants who lacked day-90 outcome data and analysed only moderate-severity TBI cases; thus, our sample omits the more prevalent mild injuries—also associated with post-TBI anxiety and depression—and the severe injuries – which are most costly in terms of monetary and non-monetary costs for the patient, patients’ family and society)[43,60].

Several strengths of our cost-effectiveness analysis may be highlighted, including that data was retrieved from a clinical trial conducted in a single country (and thus, challenges regarding details of patient pathway specific to a trial conducted in multiple countries are eliminated), and that scales used to quantify the cost per one point improvement are relevant for this group of patients as anxiety and depression are among the most common post-TBI sequalae. Although this is the first cost-effectiveness analysis among patients with moderate TBI, additional cost-effectiveness analyses covering patients with different levels of severity and tackling some of the limitations of our study (sample size or usage of health-related quality of life) should be conducted to provide coverage for TBI patients suffering from mild (which are the most common form) or severe cases.

## CONCLUSION

Our study suggests that Cerebrolysin may be cost-effective in patients with moderate-severe TBI, underscoring the importance of adopting a holistic view in managing TBI patients.

## Data Availability

The data presented in this study were obtained from the CAPTAIN II study and are available upon reasonable request from the corresponding author.
